# Exploring The Impact of Positive Behaviour Support Plans on Adult Acute Mental Health Staff Practice

**DOI:** 10.1192/j.eurpsy.2024.1245

**Published:** 2024-08-27

**Authors:** J. M. Plant, J. Beezhold

**Affiliations:** Norfolk and Suffolk NHS Foundation Trust, Norfolk, United Kingdom

## Abstract

**Introduction:**

There is a national movement to reduce restrictive interventions due to the harm and distress they can cause which has been reflected in NHS trust policies and practices. NHS trust policies state that all in-patients who may require restrictive interventions must have a Positive Behaviour Support Plan (PBSP) based on a functional analysis of what drives and triggers their behaviour. A PBSP is intended to facilitate understanding and help manage behaviours that challenge by teaching new skills and ways to communicate a person’s needs. Previous research on the use of PBSPs on adult acute mental health wards is limited but research on PICU wards has shown PBSPs have not been implemented into mental health care as intended.

**Objectives:**

Trust policies identify that PBSPs should be implemented to reduce the use of restrictive interventions. However, it is unknown whether PBSPs are being used as part of routine practice on the acute mental health ward. The degree to which staff are aware of patients PBSPs and how they use them to guide their practice is unclear. The service evaluation aims to understand the perspectives, attitudes, and experiences of staff who are responsible for using and implementing PBSPs on the ward. The evaluation aims to investigate how PBSP informs practice and to identify the barriers and facilitators to implementing PBSPs on the ward.

**Methods:**

A volunteer sample of clinical staff members (including Doctors, Nurses, Psychotherapists, Occupational Therapists, and Clinical Support Workers) who are responsible for implementing PBSPs on an acute mental health ward in the East of England took part in a focus group which lasted up to an hour. There were four focus groups with between two and four participants per group. A total of thirteen staff members participated in the focus groups. The focus groups lasted up to one hour and were guided by a topic guide. Two members of the project team facilitated the group. Focus groups were audio recorded.

**Results:**

Thematic synthesis will be the overarching approach used to synthesise the qualitative data from the focus groups. The audio recordings will be transcribed. Analysis will be conducted on a within-case basis prior to cross-case analysis aimed to identify common themes. Two evaluators will work together to code, analyse, and synthesise the extracted data.

**Conclusions:**

Based on the results, training may be developed to improve the understanding and implementation of the PBSPs on the ward. The findings may also result in changes to the way PBSPs are used. The results will be presented to the trust chief executives and used to inform how to best support individuals who may be at risk of requiring restrictive interventions.

**Disclosure of Interest:**

None Declared

